# Functional outcomes before and after implant removal in patients with posttraumatic shoulder stiffness and healed proximal humerus fractures: does implant material (PEEK vs. titanium) have an impact? – a pilot study

**DOI:** 10.1186/s12891-022-05061-x

**Published:** 2022-01-27

**Authors:** E. Fleischhacker, C. M. Sprecher, S. Milz, M. M. Saller, J. Gleich, G. Siebenbürger, T. Helfen, W. Böcker, B. Ockert

**Affiliations:** 1 Department of Orthopaedics and Trauma Surgery, Musculoskeletal University Center Munich (MUM), University Hospital, LMU Munich, Marchioninistr. 15, 81377 Munich, Germany; 2 AO Research Institute Davos, Davos, Switzerland; 3 Anatomische Anstalt der Ludwig-Maximilians-Universität, Munich, Germany

**Keywords:** Humerus fracture, Osteosynthesis, Implant removal, Posttraumatic shoulder stiffness, Implant material

## Abstract

**Background:**

Posttraumatic shoulder stiffness remains a problem after proximal humerus fracture (PHF) despite good healing rates. The aim of this pilot study was to determine whether the implant material and overlying soft tissue have an effect on shoulder range of motion (ROM) before and after implant removal (IR).

**Methods:**

16 patients (mean age 55.2 ± 15.3 (SD) years; 62.5% female) were included who underwent operative treatment with locking plates of either carbon fiber reinforced Polyetheretherketone (PEEK) (PEEKPower® humeral fracture plate, Arthrex, Naples, Florida, USA, *n* = 8) or titanium alloy (Ti) (Philos®, DePuy Synthes, Johnson & Johnson Medical, Raynham, Massachusetts, USA, *n* = 8) for PHF. All patients presented with a limited ROM and persistent pain in everyday life after the fracture had healed, whereupon IR was indicated. ROM before and after IR were compared as well as the Constant Score (CS) and the CS compared to the contralateral shoulder (%CS) 1 year after IR.

**Results:**

In group PEEK, elevation was 116.3° ± 19.2° pre- and 129.4° ± 23.7° post-IR (*p* = 0.027). External rotation was 35.0° ± 7.6° pre- and 50.6° ± 21.8° post-IR (*p* = 0.041). External rotation with the humerus abducted 90° was 38.8° ± 18.1° pre- and 52.5° ± 25.5° post-IR (*p* = 0.024). In group Ti, elevation was 110.0° ± 34.6° pre- and 133.8° ± 31.1° post-IR (*p* = 0.011). External rotation with the humerus at rest was 33.8° ± 23.1° pre- and 48.8° ± 18.7° post-IR (*p* = 0.048). External rotation with the humerus abducted 90° was 40.0° ± 31.6° pre- and 52.5° ± 22.5° post-IR (*p* = 0.011). Comparison of the two implant materials showed no significant difference. The overall CS was 90.3 ± 8.8, the %CS was 91.8% ± 14.7%.

**Conclusion:**

There was no significant difference in ROM, CS and %CS with respect to plate materials, although lower cell adhesion is reported for the hydrophobic PEEK. However, all patients showed improved functional outcomes after IR in this pilot study. In patients with shoulder stiffness following locked plating for PHF, implants should be removed and open arthrolysis should be performed, independently from the hardware material.

**Level of evidence:**

II

**Supplementary Information:**

The online version contains supplementary material available at 10.1186/s12891-022-05061-x.

## Background

Posttraumatic shoulder stiffness is a major problem after proximal humerus fracture [1]. Although the third most common fracture in human individuals heals well with both surgical and conservative therapy, the clinical outcome in many cases is characterized by severe limitation of shoulder motion [1–5]. Some studies report a loss of more than 50%, which occurs despite regular bone healing on radiographs [1]. In young patients, it often leads to a long period of inability to work, and in older patients, long-term impaired self-care may be the consequence [6, 7].

In contrast to idiopathic shoulder stiffness, where the cause is attributed primarily to inflammation of the joint capsule, the origin of posttraumatic shoulder stiffness remains largely unclear [3]. Posttraumatic shoulder stiffness has received little attention in literature over the past years. The extent to which the joint capsule itself or periarticular adhesions after proximal humerus fracture are causative for the movement disorder remains largely unknown [2, 8, 9]. Similarly, it is unclear whether specific implants have a discernable effect on shoulder mobility after surgical treatment [10, 11]. In addition to mechanical impediments (e.g. subacromial impingement if the plate is placed too high), many patients show distinctive adhesions between the implant and the overlying tissue layers [10–12].

After bony consolidation of the fracture, many of these plates are removed [13, 14]. Material removal accounts for up to 30% of all elective orthopedic trauma surgery procedures [13]. Common indications are pain and persistent limitation of motion, which cannot be improved by physiotherapy [13, 15, 16]. It has been shown that by removing the plate and the soft (fibrotic) tissue that has grown around it, the range of motion can be significantly improved [13].

Implants for internal fixation of proximal humerus fractures are made of titanium alloy, steel and carbon fiber reinforced polyetheretherketone (PEEK). Due to some disadvantages, such as corrosion, allergic reactions and infections, steel has lost its importance and titanium alloy is now mostly used [17]. In recent years, however, PEEK has become increasingly important. The chemical structure of this hydrophobic, polyaromatic ketone causes stability at high temperatures (over 300 °C), gives resistance to chemical and radiation damage, and higher strength (per mass) than many metals [18, 19]. Therefore, it is also inert and due to its radiolucency offers advantages during fracture reduction [18, 19]. Furthermore clinicians describe haptic and visual differences of the soft tissue that forms around the implants. The soft tissue over titanium alloy for example forms a firm bond (i.e. is adhesive to the surface) with the implant, whereas the soft tissue over steel tends to form a capsule [10]. PEEK was said to form fewer adhesions with the surrounding soft tissue due to its hydrophobic properties [20]. According to the authors’ clinical experience, the soft tissue over PEEK implants seems to behave more like that over titanium alloys insofar as it also forms a firm bond with the implant. Since PEEK, like titanium, is inert, this does not seem surprising at first glance, but it is because of the hydrophobicity and the poor cell-adhesive properties described in the literature [20]. Besides, the soft tissue over PEEK plates appears thicker and stiffer, but valid data are lacking. The impact of these differences on implant removal and range of motion remains unclear.

Based on the aforementioned aspects, the aim of this pilot study was to investigate whether the different material properties influence the range of shoulder motion before and after implant removal at the proximal humerus. The hypothesis was that there are differences in the benefit of implant removal in terms of postoperative range of motion between different implant materials.

## Materials and methods

After approval by the local ethics committee of the Ludwig-Maximilians-University (LMU) Munich, Germany, this study was accomplished according to the CONSORT (CONsolidated Standards of Reporting Trials) 2010 guideline in compliance with the Helsinki Declaration and registered in DRKS (registration date 07/12/2021, registration number DRKS00020128, https://www.drks.de/drks_web/navigate.do?navigationId=trial. HTML&TRIAL_ID=DRKS00020128). All patients consented to participate in this study after detailed informed consent.

**Fig. 1:**
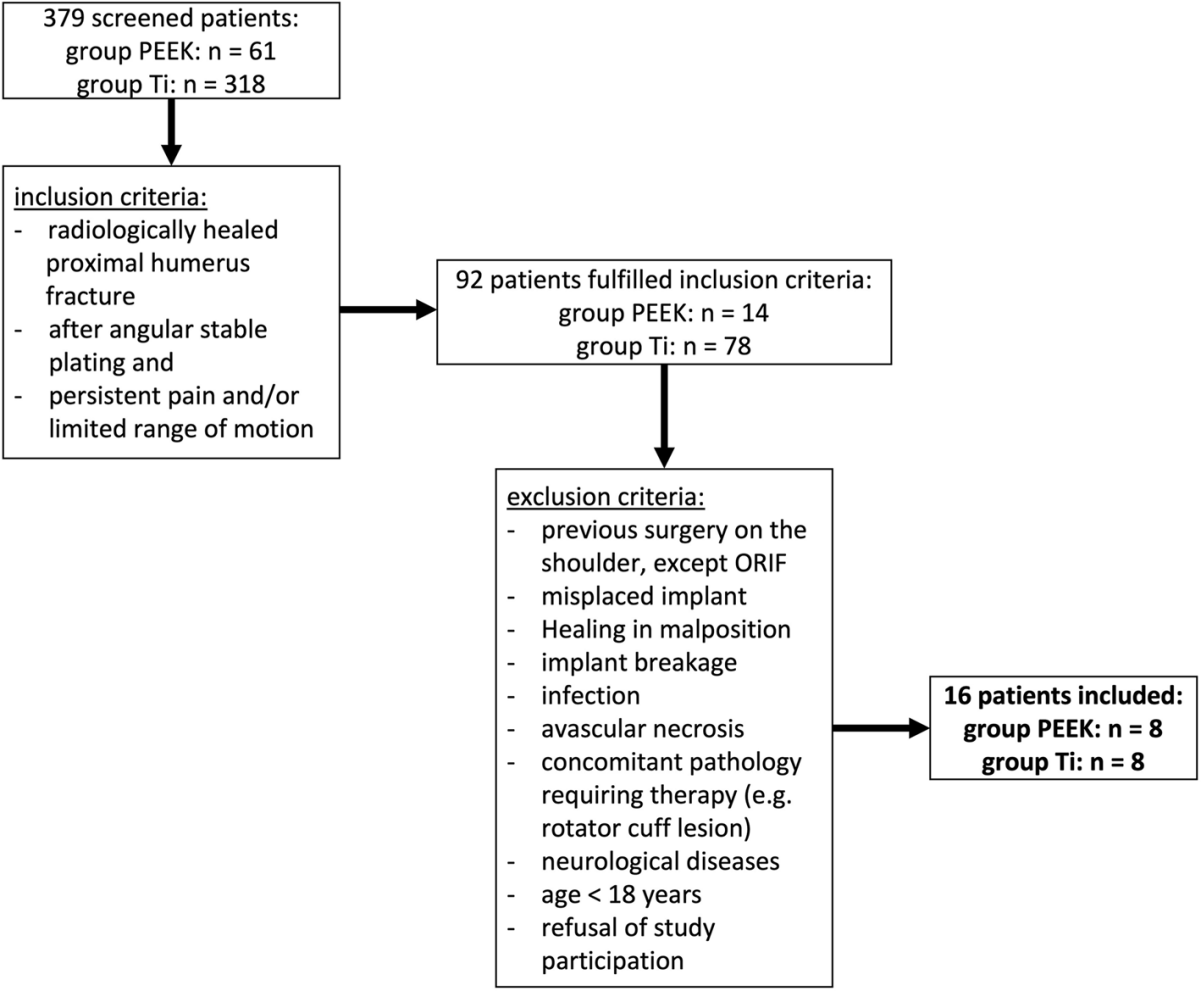
Flowchart detailing the inclusion of patients for final analysis. All in all 379 patients (PEEK *n* = 61, Ti *n* = 318) were studied of whom 92 (PEEK *n* = 14, Ti *n* = 78) patients met the inclusion criteria. After applying the exclusion criteria, 16 patients could be included in the study

Patients who requested implant removal of a previously surgically treated proximal humerus fracture due to persistent complaints such as pain and persisting limitation of range of motion in their shoulder were enrolled (Fig. 1). Primary fracture treatment by open reduction and internal fixation with plates either of PEEK (PEEKPower® humeral fracture plate, Arthrex, Naples, Florida, USA) or titanium alloy (Ti) (Philos®, DePuy Synthes, Johnson & Johnson Medical, Raynham, Massachusetts, USA), took place between June 2015 and August 2019. Before implant removal, fracture healing was radiologically ensured by X-ray of the shoulder in two planes or in case of doubt, by computer tomography (CT). The indication for implant removal was given in the case of persistent movement impairment and/or pain. On average, implant removal was performed 13.7 ± 5.6 months after primary fracture treatment. All patients were operated on both times through the same deltopectoral approach. The patients baseline characteristics are presented as supplementary data.

The passive range of motion of elevation/adduction, external/internal rotation with the humerus in neutral position and 90° flexion in the elbow joint as well as external/internal rotation with the humerus abducted by 90° and 90° flexion in the elbow joint were documented according to the neutral zero method. They were each recorded intraoperatively on the anesthetized patient before and after implant removal and extraarticular arthrolysis.

To evaluate the sustainability of the intraoperative outcome, the absolute Constant Score (CS) and the relative Constant Score compared to the contralateral side (%CS) were collected in all patients at the one-year routine follow-up after implant removal.

### Implants

**Fig. 2:**
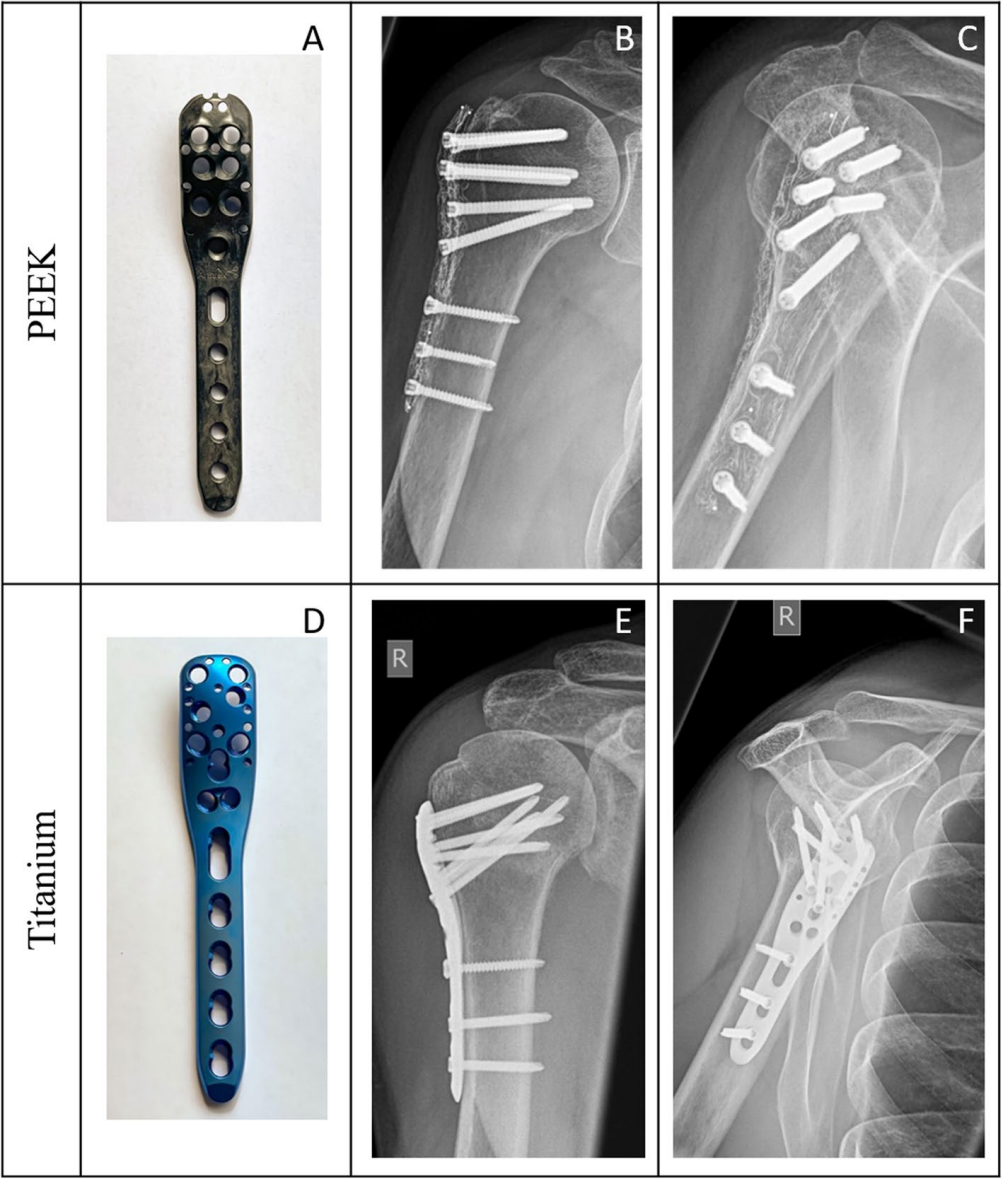
Photographs and radiographs (a.p. and lateral view) of a PEEK (**A** – **C**) and a titanium (**D** – **F**) plate. All radiographs show the last routine x-rays before implant removal. PEEK (**A** – **C**): female patient, 63 years at trauma, fracture classification according to AO 11-B1. Titanium (**D** – **F**): male patient, 45 years at trauma, fracture classification according to AO 11-B1

The implants used in this study were the PEEKPower® humeral fracture plate (Arthrex, Naples, Florida, USA) made of carbonfiber reinforced Polyetheretherketone (PEEK) (Fig. 2 (A – C)) and the proximal humerus interlocking system (Philos®, DePuy Synthes, Johnson & Johnson Medical, Raynham, Massachusetts, USA) made of titanium alloy (Ti) (Fig. 2 (D – F)).

Both implants are anatomically designed locking plates and use 3.5 mm diameter titanium alloy locking screws. In contrast to the Philos®, the PEEKPower® humeral fracture plate offers the possibility of polyaxial locking. In this case, the head of the screws can be secured in the screw hole of the plate at an angle of ±12°. For this, the head thread of the titanium alloy screw cuts a thread into the PEEKPower humerus fracture plate when it is inserted. The plate thread in the Philos®, on the other hand, is milled by the manufacturer. In addition, the plate designs differ in the arrangement and number of screw holes in the wider area, which is fixed to the humeral head (Fig. 2). Common features, however, are the presence of an oblong hole in the shaft area and the possibility of refixation of the tendons of the rotator cuff via small holes at the edge of the plates (Fig. 2).

### Surgical procedure of implant removal

**Fig. 3:**
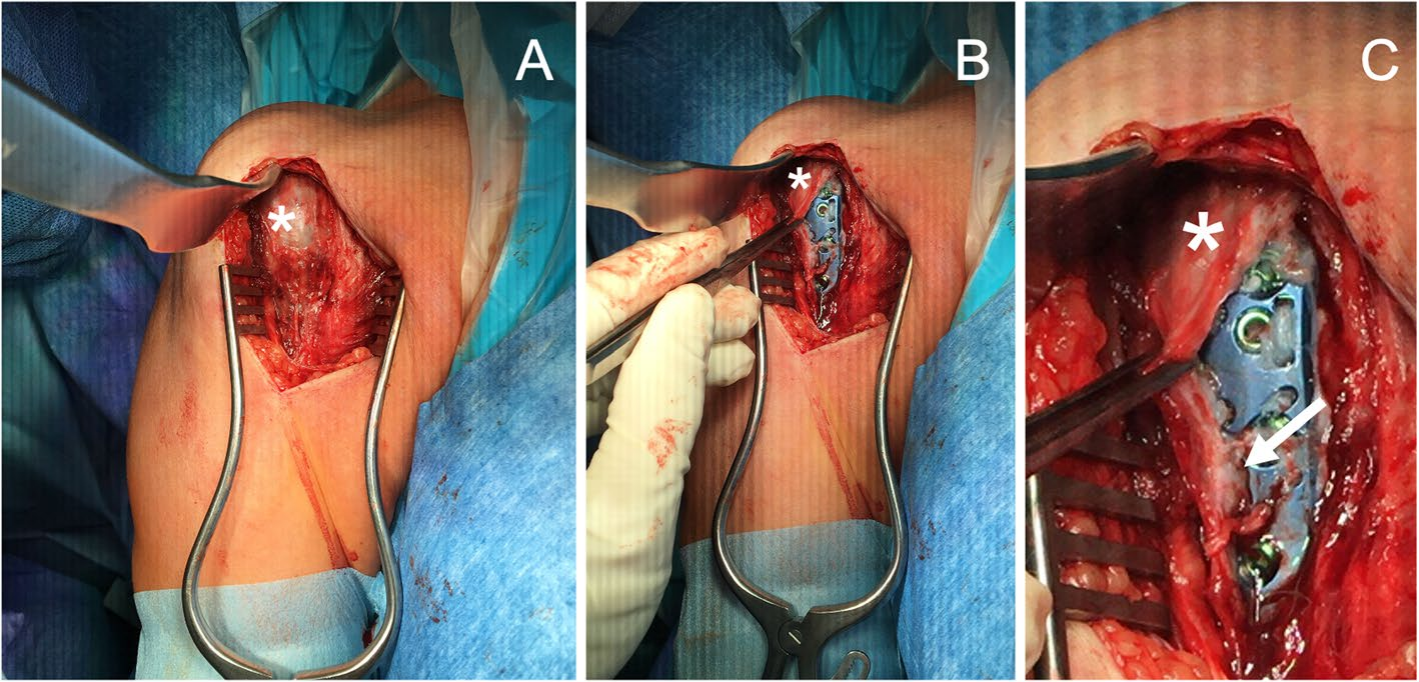
Intraoperative view of the connective tissue layer (*) that forms over the plates, here shown on the example of a titanium alloy plate. **A** shows the closed tissue layer (*), **B** and **C** a view after partial preparation of the plate. At **C**, the thickness of the soft tissue (*) becomes visible: It is an approx. 2–3 mm thick little vascularized, light-colored tissue layer. The white arrow marks the adhesions of the tissue with the implant

For implant removal, the patients were placed in beach chair position. All patients received general anesthesia and the arm to be operated on was exposed. The skin incision was made in the area of the old scar after disinfection and sterile draping as well as survey of the range of motion according to the above-mentioned scheme. Significant widening or additional incision was not necessary in any of the cases. After blunt dissection to the muscles, dissection to the bone was performed through the infraclavicular fossa between the bellies of the deltoid and pectoralis major muscles. The soft tissue layer that had formed over all plates (Fig. 3, asterisk) was completely resected during extraarticular arthrolysis. After that the plate and screws could be removed. Screws whose heads were damaged could be extracted with the help of a left-hand thread. The screw heads whose threads had made firm connections with the plate were drilled out using carbide drills. Afterwards the threads were removed by milling over them with a hollow cutter. After extensive irrigation, the wound was closed layer by layer. Then, before ending the anesthesia, the range of motion was examined again according to the above scheme. Postoperatively, all patients were allowed to move the operated shoulder without any restrictions and were given physical therapy guidance to do so; they were advised to avoid peak loads and contact sports for 6 weeks.

### Statistics

Statistical analysis was performed using SPSS Statistics, Version 26 (IBM Corp. Released 2020. Amonk, NY, USA​). After testing for normal distribution using the Shapiro-Wilk test a t-test or a Mann-Whitney Test was performed to compare the two implant materials. The correlation between the range of motion before and after implant removal and excision of the overlying soft tissue, was calculated using Pearson’s and Spearman’s correlation coefficients. A Wilcoxon test was used to compare the two time points pre-IR and post-IR within the implant groups. Data are reported as mean ± standard deviation, for categorical data as absolute frequency with percentage distribution. The significance level was set at *p* < 0.05.

## Results

Each group (PEEK and Ti) included 8 patients (Fig. 1). They were all included in the study before implant removal but after primary fracture treatment. The mean age was 55.2 ± 13.5 years and 62.5% were female. Implant removal was indicated at the earliest after 7.2 months and when fracture healing was radiologically confirmed. Radiographs before implant removal showed a regular plate position in all patients. Radiologically, none of the implants was positioned too high (< 5 mm distal to the apex of the tuberculum majus).

Problems with implant removal are reported in two patients whose fractures were retained using Philos® titanium alloy plates. Due to a tight connection between screw and plate, these could not be unscrewed in the conventional way, but had to be overdrilled. In patients whose fracture had been fixed with a PEEK plate, there were no implant related problems intraoperatively during implant removal.

All patients in group PEEK and in group Ti benefited from implant removal in all dimensions of motion. The graphical representation of the comparison between the implant materials and between before and after implant removal can be seen in Fig. 3. The exact numerical values as well as the differences (delta) are shown in Table 1 (PEEK) and 2 (Ti).

**Table 1:** Differences in range of motion in patients after fracture retention with PEEK-plates before and after implant removal and excision of the overlying soft tissue (IR). The statistical analysis was performed using the Wilcoxon test

Range of Motion	Before IR	After IR	Difference (Δ)	p
elevation	116.3° ± 19.2°	129.4° ± 23.7°	13.1°	0.027
adduction	15.0° ± 10.7°	23.1° ± 16.2°	8.1°	0.041
external rotation humerus 0° abducted	35.0° ± 7.6°	50.6° ± 21.8°	12.5°	0.041
internal rotation humerus 0° abducted	58.8° ± 33.1°	71.3° ± 38.3°	15.6°	0.041
external rotation humerus 90° abducted	38.8° ± 18.1°	52.5° ± 25.5°	13.7°	0.024
internal rotation humerus 90° abducted	53.8° ± 32.5°	67.5° ± 30.1°	13.7°	0.041

**Fig. 4:**
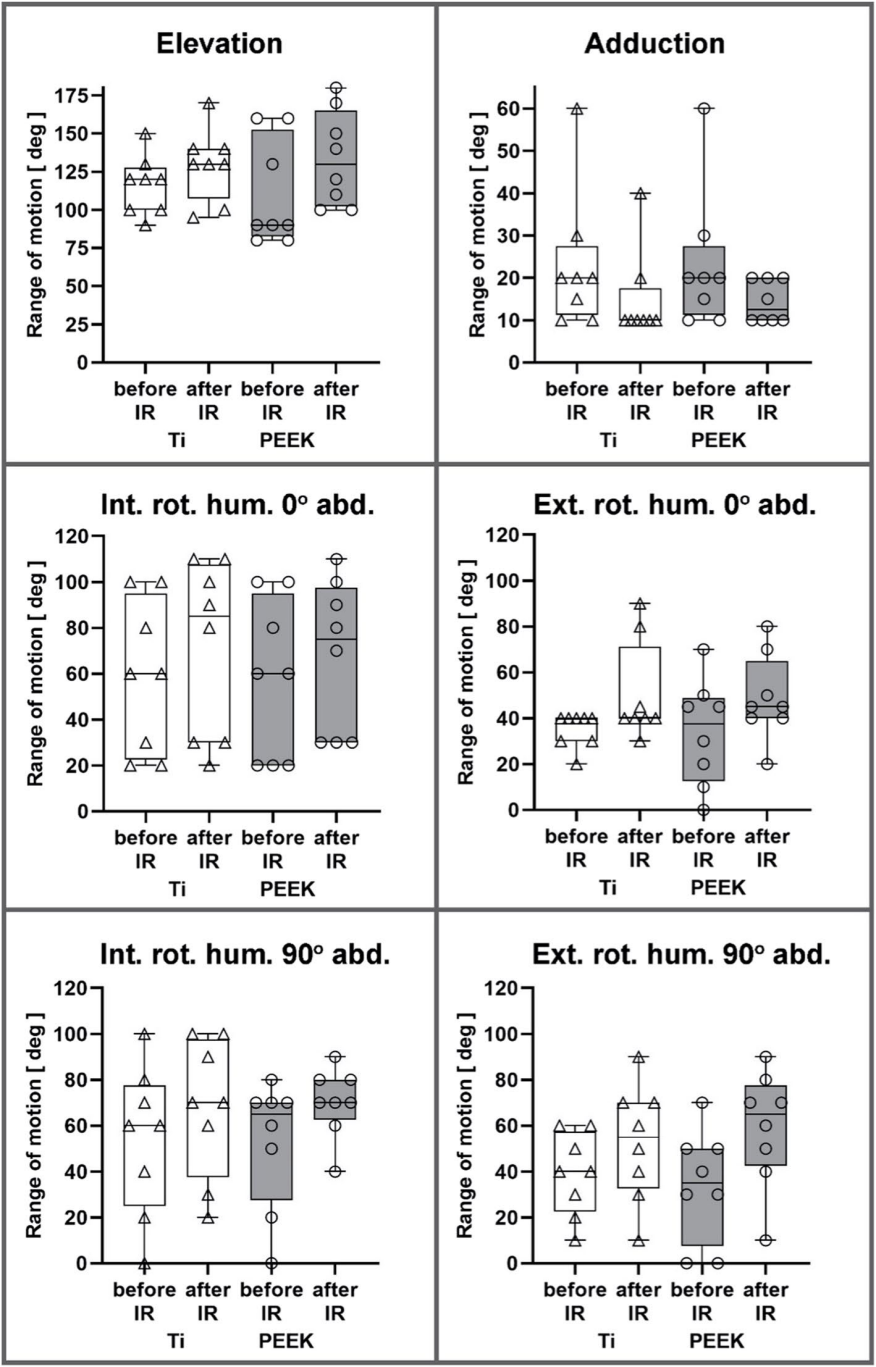
Comparison of the range of motion for the individual movement dimensions within group PEEK and Ti before and after removal of the implants and the overlying soft tissue. In all dimensions there is an improvement from before to after implant removal

In the statistical evaluation patients in group PEEK showed differences in range of motion in all dimensions in the Wilcoxon test (Table 1) before and after implant removal (Fig. 4). In group Ti, the Wilcoxon test showed significant differences in elevation, internal and external rotation with the humerus at rest and internal rotation with the humerus abducted by 90° after implant removal and excision of the surrounding soft tissue (Table 2, Fig. 4). In contrast, adduction and external rotation with the humerus abducted 90° showed no significant differences before and after implant removal (Table 2, Fig. 4).

**Table 2:** Differences in range of motion in patients after fracture retention with titanium alloy plates before and after implant removal and excision of the overlying soft tissue (IR). The statistical analysis was performed using the Wilcoxon test

Motion	Before IR	After IR	Difference (Δ)	p
elevation	110.0° ± 34.6°	133.8° ± 31.1°	23.8°	0.011
adduction	14.4° ± 5.0°	18.8° ± 3.5°	4.4°	0.059
external rotation humerus 0° abducted	33.8° ± 23.1°	48.8° ± 18.7°	10.0°	0.048
internal rotation humerus 0° abducted	57.5° ± 34.5°	67.5° ± 33.3°	10.0°	0.011
external rotation humerus 90° abducted	33.8° ± 24.5°	58.8° ± 25.3°	25.0°	0.011
internal rotation humerus 90° abducted	52.5° ± 28.2°	70.0° ± 15.1°	12.5°	0.026

**Fig. 5:**
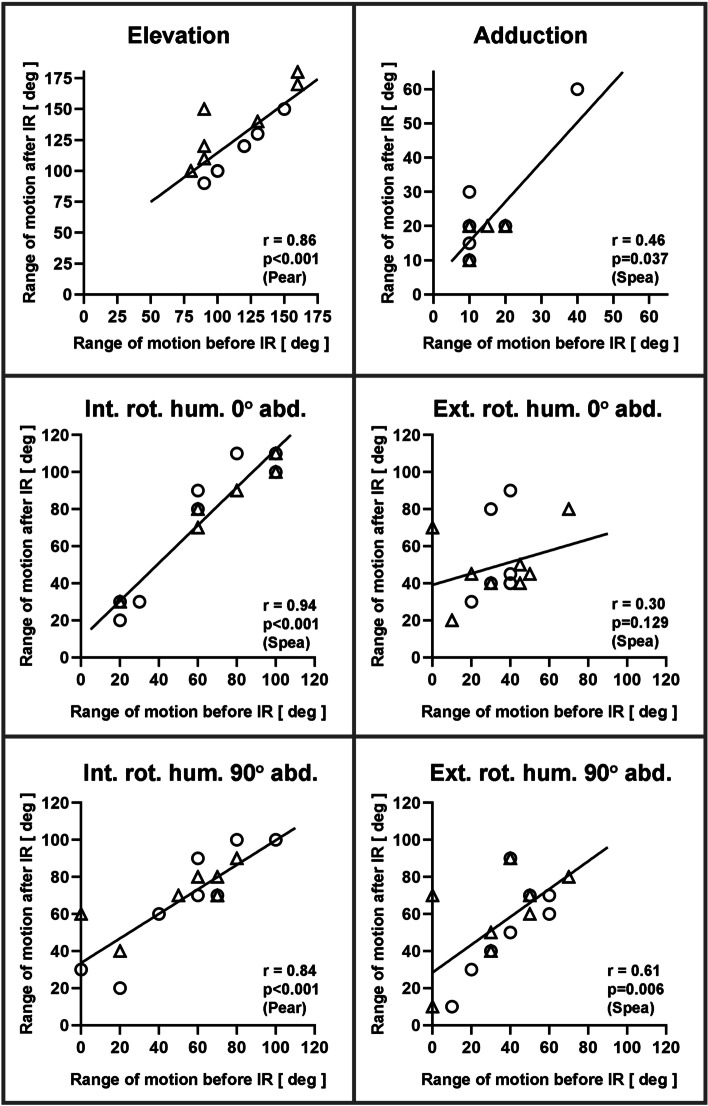
Correlation of range of motion before and after implant removal (IR). The circles symbolize patients with PEEK implants and the triangles those with titanium alloy implants. Data were correlated using Pearson’s (Pear) and Spearman’s (Spea) correlation coefficients, respectively. With r > 0.5, a strong correlation can be shown, with r > 0.3 there is a medium correlation

Comparison of the range of motion between the two implant materials using the t-test or Mann-Whitney test showed no statistically significant difference in either the results before or after implant removal and excision of the overlying soft tissue. Calculation of the correlation using Pearson’s and Spearman’s correlation coefficient shows a strong correlation (r > 0.5) of the pre- and post-IR values for elevation, internal rotation at 0° abducted and 90° abducted humerus as well as external rotation at 90° abducted humerus (Fig. 5). An intermediate correlation (r > 0.3) was found for adduction and external rotation at 0° abducted humerus (Fig. 5).

**Fig. 6:**
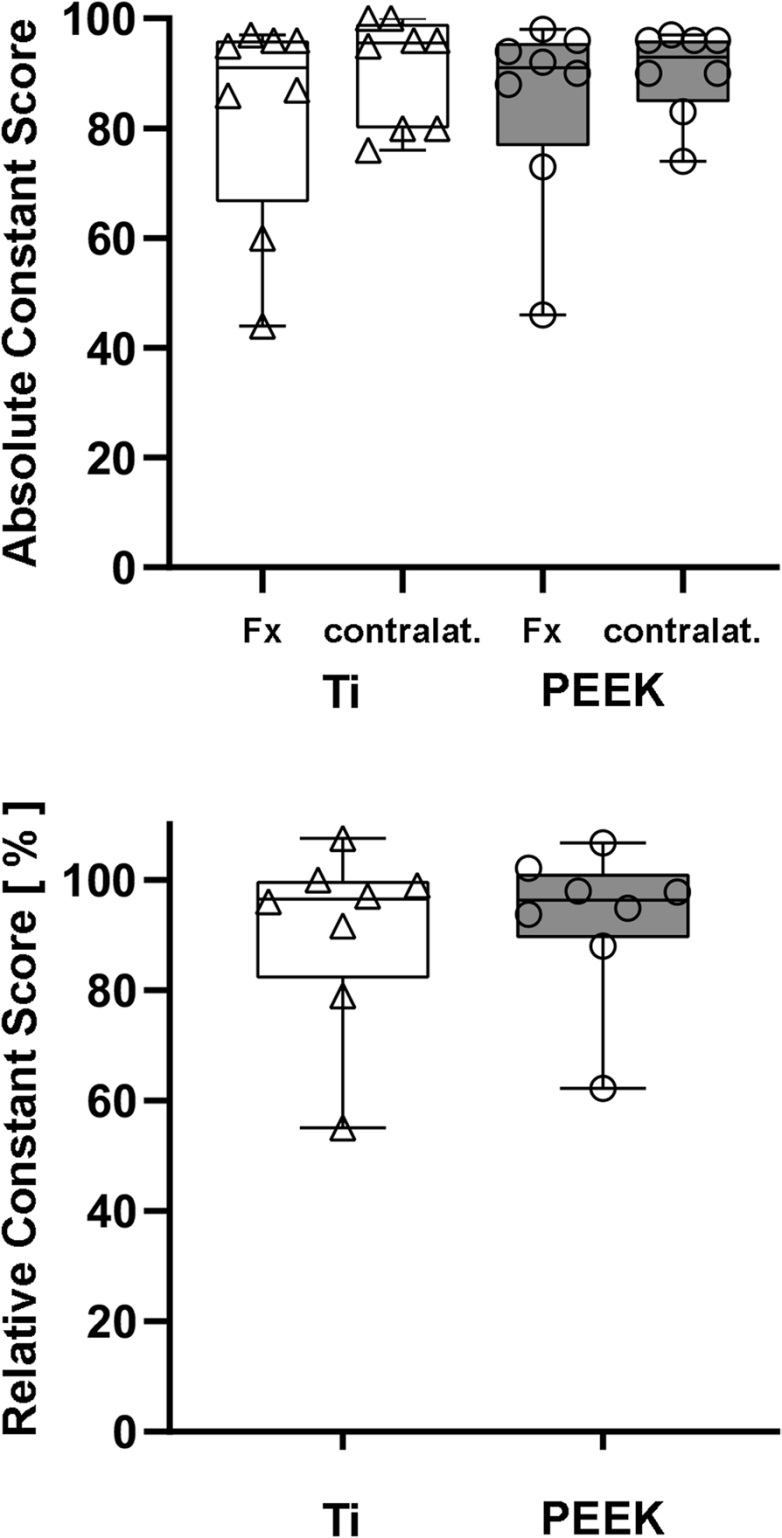
Absolute constant score (CS) and relative constant score of the injured shoulder (fx) compared to the contralateral (contralat.) shoulder one year after implant removal in group PEEK and group Ti. There is no statistically significant difference regarding the two implant materials

At the one-year follow-up, the overall CS was 90.3 ± 8.8 (out of 100 points) and the overall %CS was 91.8% ± 14.7%. The mean CS in group PEEK was 84.6 ± 18.0, in group Ti it was 82.6 ± 19.8. Compared to the contralateral side, the %CS was 92.9% ± 13.6% in group PEEK and 90.6% ± 16.6% in group Ti. In the statistical evaluation, there was no significant difference between the implant materials (Fig. 6). 87.5% of all patients subjectively reported a significant improvement in range of motion and reduction of pain after implant removal and would do it again or recommend it.

## Discussion

This work demonstrated that regardless of the implant material, range of motion for elevation, internal and external rotation with the humerus at rest, and internal rotation with the humerus abducted 90° can be significantly improved after implant removal and excision of the overlying soft tissue at the proximal humerus. In patients whose fracture had been retained using a PEEK plate, adduction and external rotation with the humerus abducted 90° also showed significant improvement. In addition, evaluation of the CS and %CS showed a good functional outcome even 1 year after implant removal.

These results confirm previous studies in which patients with reduced shoulder function after plate osteosynthesis of a proximal humerus fracture had a better functional outcome after implant removal [13, 14, 21–23]. Acklin et al. showed that the Constant Score improved significantly from pre- to postoperative in 20 patients with persistent limited range of motion, after elective implant removal at the proximal humerus. In this study, all patients were treated using the Philos® plates; no statement is made about the implant material [22]. Similarly, Katthagen et al. demonstrated that abduction improved significantly after open implant removal in all 9 patients included. However, the main focus of this study was the evaluation of a new arthroscopic implant removal technique. Here, significant improvement in anteversion was achieved after arthroscopic implant removal [14]. Waszczykowski et al. highlighted in their publication that in addition to implant removal, excision of the soft tissue overlying the plates can also be attributed positive effects in postoperative improvement of range of motion. However, no statement is made about implant materials in this study either [23]. In this previous work, especially external rotation with the humerus at rest was improved by releasing the adhesions over the plate, [23] whereas the data presented in that manuscript also show an improvement in external rotation with the humerus abducted at 90° for PEEK plates. Although only small patient cohorts were evaluated in all publications, it can be stated that in patients with persistent limited range of motion after plate osteosynthesis of a proximal humerus fracture, implant removal and extraarticular arthrolysis can improve the range of motion. These results are, at least in Germany, also of economic relevance, since it makes a difference in the assessment of the pension/compensation payment whether the patient can lift the arm by 60°, 90° or more than 120°. Thus, between less than 90° and more than 120° elevation is 10% when determining the degree of disability or when assessing the reduction in incapacity for work for the public pension insurance [24]. Private accident insurers even estimate a disability of 6/20 arm value, i.e. 30%, for an elevation less than 60° [25].

Besides it is important to note that in the small cohort studied here, in two patients in group Ti problems with screw removal occurred because their head threads had formed a tight connection with the plates. This resulted in a longer surgical procedure and therefore also anesthesia time as well as an increased risk of intraoperative fractures due to the increased use of force [26]. This type of complication could only occur because the plate and screws were made of metals. With PEEK-metal pairings, making such a tight connection between the head thread and the plate is not possible due to the material properties.

The limitations of this study are due to the small number of patients and the short study period, besides a certain selection bias cannot be excluded. This makes the study prone to second-order errors and reduces the statistical power. Furthermore, no Constant scores were collected preoperatively for comparison.

Regarding the differences between the implant materials, the hypothesis had to be rejected as no statistically significant difference was found. It is assumed that the hydrophobic surface properties of PEEK are responsible for the poor cell adhesion properties, which is an advantage for implants whose integration into the tissue is not wanted, such as osteosynthesis plates. However, the authors’ clinical experience contradicts these literature statements, and the results of this pilot study also show that PEEK does not cause lower adhesion with the surrounding soft tissue than titanium alloys. At least this effect is not reflected in the functional outcomes of the patients. Nevertheless, this peri-implant soft tissue provides an interesting basis for future studies and should be further investigated as it could be one possible causative factor for post-traumatic shoulder stiffness.

To the best of our knowledge this was the first study to investigate and compare different implant materials in patients with persistent limited range of motion after plate osteosynthesis of a proximal humerus fracture, even though finally there was no significant difference between the implant materials regarding pre- and post-IR range of motion.

## Conclusion

There is no significant difference in range of shoulder motion following implant removal and excision of the overlying soft tissue with respect to plate materials, although lower cell adhesion has been reported in the literature for hydrophobic PEEK. However, all patients showed improved functional outcomes after implant removal and there was an intermediate to strong correlation between pre- and intraoperatively measured range of motion. Therefore, in patients with shoulder stiffness following locked plating for proximal humeral fractures, regardless of the implant material, the authors make a cautious recommendation for implant removal and excision of the peri-implant soft tissue. This pilot study gives us an indication that in the search for the etiology of posttraumatic shoulder stiffness, the implants and overlying soft tissue should not be ignored and require further investigation. It shows that one possible reason for postoperative limited range of motion may be found in this tissue.

## Supplementary Information

**Additional file 1.**

## Data Availability

The datasets used and/or analysed during the current study are available from the corresponding author on reasonable request.

## Abbreviations

PHF: Proximal humerus fracture
ROM: Range of motion
PEEK: Carbonfiber reinforced Polyetheretherketone
SD: Standard Deviation
Ti: Titanium
CS: Constant Score
%CS: Relative Constant Score compared to the contralateral shoulder
CT: Computer tomography
a.p.: Anterior-posterior
deg: Degree
IR: Implant removal and excision of overlying soft tissue
Int. rot. Hum. 0° abd.: Internal rotation with the humerus 0° abducted
Ext. rot. Hum. 0° abd.: External rotation with the humerus 0° abducted
Int. rot. Hum. 90° abd.: Internal rotation with the humerus 90° abducted
Ext. rot. Hum. 90° abd.: External rotation with the humerus 90° abducted
Pear: Pearson’s correlation coefficients
Spea: Spearman’s correlation coefficients
Fx: Injured shoulder
contralat.: Contralateral shoulder
