# Supplementary material for: Functional outcomes before and after implant removal in patients with posttraumatic shoulder stiffness and healed proximal humerus fractures: does implant material (PEEK vs. titanium) have an impact? – a pilot study
Source: BMC Musculoskelet Disord. 2022 Jan 27;23:95. doi: 10.1186/s12891-022-05061-x (PMC8796509; doi:10.1186/s12891-022-05061-x)
Supplement: Supplementary file 1 — Additional file 1. [file 12891_2022_5061_MOESM1_ESM.docx]

**Supplementary Data**

Supplementary Data: Overview of patient characteristics in the two study groups. IR = Implant removal

| Patient | Material | Age (range) at trauma [years] | months between trauma and IR | Gender | Fracture classification (AO) | ASA classification |
| --- | --- | --- | --- | --- | --- | --- |
| 101 | PEEK | 65 | 15.5 | female | 11-B2 | 2 |
| 102 | PEEK | 29 | 8.4 | female | 11-A2.2 | 1 |
| 103 | PEEK | 76 | 11.4 | female | 11-B2 | 3 |
| 104 | PEEK | 65 | 11.2 | female | 11-A3 | 3 |
| 105 | PEEK | 76 | 11.9 | female | 11-B1 | 2 |
| 106 | PEEK | 60 | 13.5 | female | 11-A3 | 2 |
| 107 | PEEK | 63 | 13.8 | female | 11-B1 | 2 |
| 108 | PEEK | 35 | 7.2 | female | 11-A3 | 2 |
| 001 | titanium | 51 | 9.9 | male | 11-C3 | 3 |
| 002 | titanium | 77 | 14.3 | male | 11-C3.1 | 3 |
| 003 | titanium | 50 | 15.9 | male | 11-B1 | 2 |
| 004 | titanium | 33 | 31.3 | male | 11-A3 | 2 |
| 005 | titanium | 48 | 11.4 | male | 11-B1 | 2 |
| 006 | titanium | 45 | 11.6 | male | 11-B1.1 | 2 |
| 007 | titanium | 48 | 10.6 | female | 11-A1.2 | 1 |
| 008 | titanium | 42 | 21.9 | female | 11-A2 | 1 |
|  |  |  |  |  |  |  |
| All | *(range)* | 55.2 ± 13.5 *(29 – 77)* | 13.7 ± 5.6 *(7.2 – 31.3)* | 62.5% female |  |  |
|  | PEEK *(range)* | 58.6 ± 17.5 *(29 – 76)* | 11.6 ± 2.8 *(7.2 – 15.5)* | 100% female |  |  |
|  | titanium *(range)* | 49.3 ± 12.6 *(33 – 77)* | 15.9 ± 7.4 *(9.9 – 31.3)* | 25%  female |  |  |
